# Efficacy and safety of Ban-Lan-Gen granules in the treatment of seasonal influenza: study protocol for a randomized controlled trial

**DOI:** 10.1186/s13063-015-0645-x

**Published:** 2015-03-28

**Authors:** Zheng-tu Li, Li Li, Ting-ting Chen, Chu-yuan Li, De-qin Wang, Zi-feng Yang, Nan-shan Zhong

**Affiliations:** State Key Laboratory of Respiratory Diseases, Guangzhou Institute of Respiratory Disease, National Clinical Centre of Respiratory Disease, The First Affiliated Hospital, Guangzhou Medical University, 151 Yanjiang Xi Road, Guangzhou, 510120 China; The First Hospital of Yulin, Yuxi Da Dao Road, Yulin, 719000 China; Macau University of Science and Technology, Avenida Wai Long, Taipa, Macau, 519020 China; Hutchison Whampoa Guangzhou Baiyunshan Chinese Medicine Company Limited, 389 Shatai Bei Road, Baiyun, Guangzhou, 510515 China

**Keywords:** Ban-Lan-Gen granule, Seasonal influenza, Oseltamivir, Evidence-based clinical trial

## Abstract

**Background:**

Ban-Lan-Gen (BLG) is a traditional Chinese herbal medicine. It has been used for the prevention and treatment of virus-related respiratory diseases such as influenza virus infection. BLG contains some antiviral compounds, but few evidence-based clinical studies have been conducted to assess its efficacy against influenza. We assessed the effects of BLG (including efficacy and safety) on the treatment of seasonal influenza in an evidence-based clinical trial.

**Methods/Design:**

We conducted a randomized, double-blinded, oseltamivir- and placebo-controlled, parallel-design clinical trial. A total of 177 subjects are going to be recruited after satisfying the criteria: (i) 18 to 65 years of age; (ii) illness onset within 36 h; (3) axillary temperature ≥38.0°C; and (iv) positive influenza (type A/B) virus test. Subjects will be assigned randomly into three groups in equal proportions: oseltamivir treatment, BLG granule treatment, and placebo treatment. Each group receives 5-day treatment and is followed up 1, 3, 5, 7 and 21 days later. Symptoms and patient compliance are recorded, and virus/serum viral antibodies tested. We will use the primary outcome, secondary outcome, and safety indicators to evaluate the efficacy and safety of BLG granules in the treatment of seasonal influenza.

**Discussion:**

We have described the first clinical trial for treatment using a single herb against influenza A and B viruses in China. We will hold a large-scale clinical trial to comprehensively evaluate the effectiveness and safety of BLG against influenza infection based on the results of this pilot study. And this clinical trial will serve as an example for the study of other traditional herbal medicines in evidence-based clinical trials.

**Trial registration:**

This study has been registered at ClinicalTrials.gov: NCT02232945 (3 September 2014).

**Electronic supplementary material:**

The online version of this article (doi:10.1186/s13063-015-0645-x) contains supplementary material, which is available to authorized users.

## Background

### The influenza epidemic

Influenza is an acute respiratory disease caused by highly infectious influenza viruses such as H1N1, H3N2, H5N1, H7N9, and influenza B. It has a strong capability of spreading globally, leading to adverse health effects [1]. Such respiratory diseases are usually caused by type A or type B influenza, and the symptoms include headache, muscle aches, cough, and sudden fever [2]. Infection by the pandemic (H1N1) 2009 virus emerged initially in Mexico in early 2009 and has become a global pandemic [3-5]. Now, influenza A (H1N1, H3N2 and pandemic (H1N1) 2009) and influenza B have induced co-infection worldwide, thereby causing considerable panic among general populations.

### Drug resistance in Western medicine

M2 ion channel blockers (for example, amantadine and rimantadine) and neuraminidase (NA) inhibitors (for example, oseltamivir, zanamivir, and peramivir) are commonly used for the prevention and treatment of influenza [6]. Amantadine and rimantadine, however, have been associated with neurologic toxicities and gastrointestinal side effects [7]. Moreover, overuse of these medicines has been found to result in drug-resistant strains [8]. Up to 2003, M2-resistant variants had even spread to countries where these drugs are used infrequently [9]. Pandemic (H1N1) 2009 has demonstrated resistance to amantadine and rimantadine [10,11]. Our previous study suggested that clinical isolates of H1N1 and H3N2 developed amantadine resistance at 93.1% and 100%, respectively, in Guangzhou, China [12]. NA inhibitors, in comparison with M2 ion channel blockers, are more commonly used but they (i) can also cause respiratory side effects and even death (for example, oseltamivir [13]) owing to intensive use and (ii) have a higher barrier for resistance if overused. A survey from 1999 and 2007 showed a very low level (<1%) of resistance to oseltamivir [10,14], but a survey between 2008 and 2009 reported the resistance to be ≥90% [15-18]. Resistance to zanamivir has been (albeit rarely) reported among immunodeficient patients [19,20]. Whether such antiviral agents can be used to address influenza epidemics is not clear. Furthermore, the true effectiveness of oseltamivir treatment against influenza is controversial [21].

### Traditional Chinese herbal medicine and basic research

Traditional Chinese herbal medicine (TCHM) is richly used in Chinese clinics and widely used to treat and prevent respiratory infection disease. Unlike Western medicine, TCHM has different mechanisms and targets of action and, potentially, could be used to overcome drug resistance and side effects. Therefore, exporting the new antiviral medicine from TCHM will be a new choice. For example, the starting material (shikimic acid) for the synthesis of the oseltamivir can be obtained from *Illicium verum*, which is a type of TCHM (Tamiflu™, Roche, Basel, Switzerland).

Among the many types of TCHMs, Ban-Lan-Gen (BLG) has a thousand-year history of use for the prevention and treatment of respiratory tract infections (particularly respiratory viral diseases). BLG was highlighted as a classic antiviral and anti-influenza agent in the *People’s Republic of China Pharmacopoeia* in 1979 and 2010, respectively. Additionally, BLG was one of the eight major medicines recommended by the Chinese government for the prevention and control of severe acute respiratory syndrome (SARS).

Several clinical studies have evaluated the efficacy of BLG granules on treatment of acute pharyngitis (among which, 50% are caused by viruses), infection of the upper respiratory tract, and influenza H1N1 [22,23]. However, none of those studies were evidence-based clinical trials.

The *Isatis indigotica* root (*IIR*) is the single largest component of BLG granules. Our previous studies suggested that the *Isatis indigoticaroot polysaccharide* (*IRPS*) and clemastanin B derived from *IIR* can inhibit various subtypes of the influenza virus *in vitro*. Inhibiting the hemagglutinin (HA) of the virus at its early stage of infection is an important antiviral mechanism of *IIR* [24-26]. *In vivo* experiments revealed clemastanin B to significantly inhibit pneumonia and virus proliferation in lung tissue (Zifeng Yang and Zhengtu Li, unpublished work). We also found that the lignin group (*IIR* isolated) inhibits the nuclear factor-kappa B signaling activated by the influenza virus and blocks exportation of the NP protein of the influenza virus (article in press). Other studies have also shown that indirubin (also contains *IIR*) and its derivatives have antiviral and anti-inflammatory effects in infection due to the influenza A (H5N1) virus [27].

### Study aims

Studies have suggested that BLG and its active ingredients may inhibit the influenza virus. Little concrete clinical evidence has been provided, however, for the efficacy of BLG in the treatment of seasonal influenza. We wished to evaluate the efficacy and safety of BLG granules for the treatment of seasonal influenza in an evidence-based clinical trial.

## Methods/Design

Ethical approval of the study protocol was granted from the Ethics Committee of the First Affiliated Hospital of Guangzhou Medical University (number, 2011014; Guangzhou, China), and informed consent was obtained from each participant.

### Design

**Figure 1:**
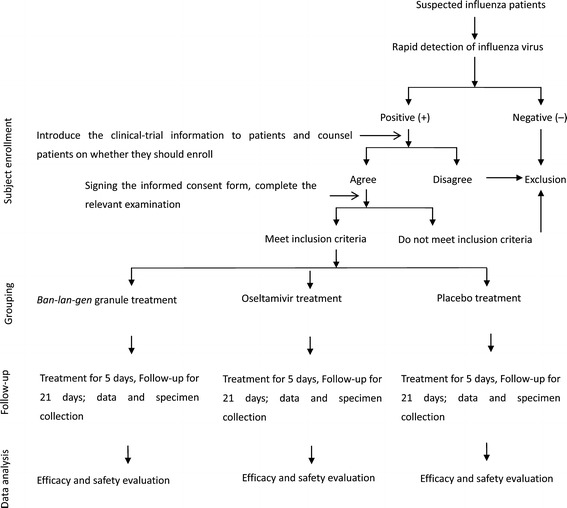
**Study flowchart.** Participants with a rapid diagnosis of influenza will be recruited, and they will be assigned randomly into three different groups. All participants will accept the 5-day treatment and 21-day follow-up period observation. The data were collected to determine the treatment efficacy and safety of Ban-Lan-Gen (BLG)*.*

This clinical study was a randomized, double-blind, double control trial that tested drugs and placebo. Noninferiority (versus positive control) and superiority (versus placebo control) trials were conducted. Patients with positive tests for the influenza virus (rapid detection of antigens to the influenza virus before enrollment, but if detection of nucleic acid of the influenza virus or cultivation of the influenza virus was inconsistent, the patient was excluded) were assigned randomly into three groups of equal proportions (1:1:1). Groups were: oseltamivir treatment (positive control), BLG granule treatment (test group) and placebo group. Patients will accept 5-day treatment and 21-day observation. Patient follow-up was undertaken on days 1, 3, 5, 7, 21 (Table 1 and Figure 1). Specific items that were collected for each follow-up period are outlined in Table 1.

**Table 1:** **Follow-up chart of treatment using ban-lan-gen granules against seasonal influenza**

**Visit cycle evaluation projects**	**Screening stage**	**Remedial period**			
	**Visit 0 day 0.5 to 0**	**Visit 1 Day 3 + 1 after administration**	**Visit 2 Day 5 + 1 after administration**	**Visit 3 Day 7 + 1 after administration**	**Visit 4 Day 21 + 7 after administration**
Acquisition of basic medical history					
Informed consent	X				
Basic conditions	X				
Symptoms	X	X	X	X	
Inclusion and exclusion criteria	X				
Safety observations					
Vital signs	X			X	
Electrocardiography	X	a	a	X	
Chest radiograph	X	a	a	a	
Laboratory inspection	X	a	a	X	
Record adverse events	X	X	X	X	X
Efficacy					
Record influenza symptoms	X	X	X	X	
Virology					
Rapid diagnostic tests	X				
Real-time PCR analyses	X	X	X	X	
Viral isolation culture	X	X	X	X	
Viral antibody titer	X				X
Other					
Grouping and giving drugs	X				
Administer drugs	X	X	X		
Distribute patient diary card	X				
Accompanying treatment	A	A	a	a	
Compliance	X	X	X	X	
Inspection results	X				X
Original record completion	X	X	X	X	X
Recover study drug				X	
Recover patient diaries				X	
CRF review, recovering and test summary					X

X, must implement; a, necessary to implement.

### Setting

Different aspects of the study activities will be conducted at different sites. For instance, patient enrollment, as well as collection of specimens and data, will be conducted in the Outpatient Department of the First Affiliated Hospital of Guangzhou Medical University. Biochemical examinations will be undertaken in the laboratories of the First Affiliated Hospital of Guangzhou Medical University. Virus testing will be carried out in the Clinical Virology Division of the State Key Laboratory of Respiratory Disease, Guangzhou, China. Data analyses will be done at Southern Medical University, Guangzhou, China.

### Number of participants and specimen collection

Recommendations from the World Health Organization for influenza-like illnesses (ILIs) were used as the basis for influenza surveillance. If a patient presents with an acute cough and fever, clinicians must be highly vigilant to test for infections caused by the influenza virus. In general, the rate of ILI cases that lead to a clinical diagnosis of influenza infection is approximately 18 to 80%, which is higher than other clinical diagnoses, for example, acute infections of the upper respiratory tract [28]. We selected adult ILI patients for inclusion into the study. The study was designed to enroll 177 cases.

Specimen collection included pharyngeal/throat swabs and blood samples. Pharyngeal/throat secretions were obtained from the upper respiratory tract of each patient [29]. Pharyngeal/throat swabs will be used to test the mRNA of the influenza virus; the virus will be cultured. Blood samples will be used to test viral antibodies.

### Study procedure

#### Staff training

Personnel who participate directly in this study will be adequately trained to ensure the safety of the patients, blinding of the study design, data quality, and adherence to the study protocol. Personnel follow guidelines for good clinical practice (GCP).

#### Recruitment, screening, and enrollment

We will follow criteria to recruit, screen, enroll and reject patients. Patients will be enrolled if they conform to all inclusion criteria. However, they will not be enrolled if they have one of the exclusion criteria, rejection criteria, or termination standards. Furthermore, it is the responsibility of participant to cooperate with the doctor’s treatment. Additionally, they should cooperate with our follow-up and provide us with information that is not beyond the ethical approval.

#### Inclusion and exclusion criteria

Inclusion criteria are patients (i) with confirmed infection by the influenza A (H1N1, H3N2) or influenza B virus according to real-time polymerase chain reaction (PCR) or viral culture; (ii) aged 18 to 65 years; axillary temperature ≥38°C; at least two constitutional symptoms (headache, chill, myalgia or fatigue) and one respiratory symptom (cough, sore throat, or rhinitis) [30,31]; (iii) illness onset within 36 h; and (iv) granting of written informed consent. Exclusion criteria are detailed in Table 2.

**Table 2:** **Inclusion and exclusion criteria**

**Inclusion criteria**	**Exclusion criteria**
(1) With confirmed infection by the influenza A (H1N1, H3N2) or influenza B virus according to real-time polymerase chain reaction (PCR) or viral culture	(1) Age <18 years or >65 years
	(2) Confirmed bronchitis, pneumonia, pleural effusion and interstitial lung disease *via* chest imaging (radiograph or CT)
(2) Aged 18 to 65 years; axillary temperature ≥38°C and at least two constitutional symptoms (headache, chill, myalgia or fatigue) and one respiratory symptom (cough, sore throat, or rhinitis)	(3) Routine screening tests of blood with leukocyte readings >10.0 × 10^9^/L or neutrophil count ≥80%
	(4) Suppurative tonsillitis or purulent sputum
(3) Illness onset within 36 h	(5) Underling primary disorders such as hematological disease, chronic obstructive pulmonary disease (FEV_1_/FVC <70%, FEV_1_/predicated value <50%; or respiratory failure or right-heart failure), hepatic disease (ALT or AST ≥ triple ULN), renal disease (serum creatinine > 2 mg/dL) or chronic congestive heart failure (NYHA III to IV)
(4) Granting of written informed consent	
	(6) Previous administration of antiviral drugs (amantadine, rimantadine, zanamivir, or oseltamivir phosphate) before disease onset and study enrollment, or administration of traditional Chinese herbal medicine or proprietary Chinese medicine. Administration of BLG granules within 1 week before disease onset
	(7) Allergy to study medication(s)
	(8) Women who are pregnant, or may possibly become pregnant, or who are lactating with a positive urine pregnant test, or with a body mass index (BMI) ≥25 kg/m^2^
	(9) The immunodeficient: malignant tumor; organ or bone-marrow transplantation; AIDS; or taken immune inhibitors during the last 3 months
	(10) Suspicion or history of alcohol/drug abuse
	(11) Participation in another clinical trial <3 months before study randomization
	(12) Acute respiratory infection, otitis, or nasosinusitis 2 weeks before study enrollment
	(13) Vaccination with the influenza vaccine within 6 months
	(14) Other reasons at the investigator’s discretion

#### Rejection criteria and termination standards

Rejection criteria were (i) not meeting inclusion or exclusion criteria; (ii) withdrawal of informed consent; (iii) not receiving follow-up care after selection; or (iv) serious violation of the program (that is, incorrect administration leading to effects that cannot be judged).

Termination standards refer to patients who meet the inclusion criteria, but must be terminated during the trail and who are included in the final statistical analyses. Termination standards are detailed in Table 3.

**Table 3:** **Termination standards**

**Criteria**	
1	The patient’s illness becomes severe, and the severity meets one of the following criteria: (i) persistent high fever for >3 days (≥39°C); (ii) Severe cough, purulent sputum, bloody sputum, or pectoralgia; (iii) rapid breathing, trouble in breathing, lip cyanosis; (iv) alternation of consciousness (that is, drowsiness, restlessness, convulsions); (v) severe vomiting, diarrhea, dehydration; (vi) imaging confirms signs of pneumonia; (vii) levels of myocardial enzymes, such as creatine kinase (CK), creatine kinase isoenzyme (CK-MB) elevate rapidly; (viii) underlying diseases are exacerbated significantly.
2	Death (including deaths due to influenza and other causes of death)
3	Antibiotic treatment is required (bacterial pneumonia, tympanitis, nasosinusitis secondary to influenza)
4	Serious events occur
5	Other health reasons

### Drugs and usage

#### Oseltamivir treatment group

The oseltamivir treatment group will receive one oseltamivir capsule, (75 mg/per capsule), twice a day [29], 30 minutes after eating, taken with two bags of analogous BLG granules.

#### Ban-Lan-Gen *treatment group*

The BLG group will receive two bags of BLG granules (10 g/per bag), twice a day (the dosage is set according to the regulations of the China Food and Drug administration*.*), 30 minutes after eating, taken with an analogous oseltamivir capsule.

#### Placebo group

The placebo group will receive BLG analogous granules, twice a day, 30 min after eating, taken with an analogous oseltamivir phosphate capsule.

The BLG granules and the BLG analogous granules were produced according to the quality requirements of the *People’s Republic of China Pharmacopoeia* (Additional files 1 and 2)*.* Furthermore, the placebo includes white powdered sugar (41.1%), brown powdered sugar (22.6%), amylum (16.43%), dextrin (10.27%), caramel syrup (1.4%), and lemon yellow (8.2%).

All drugs were packaged by Hutchisom Whampoa Guangzhou Baiyunshan Chinese Medicine Company Limited (Guangzhou, China) according to the requirements of a double-blind, double-simulation test. The drugs used in this double-blinded trial were counted and sorted by a third party (Southern Medical University, China). All drugs were in the period of validity.

Other treatments for participants have to meet the standards for using other drugs, which are axillary temperature ≥38.5°C that continues more than 4 hours and does not degrade with physical cooling or body temperature is gradually raised ≥ 39°C. When these standards are met, the symptoms are treated as indicated: fever: paracetamol; white sputum, bromhexine; and mild asthmatic symptoms, long-term theophylline. With these exceptions, no other drugs can be used. Furthermore, detailed information on the drug use will be registered in the case report form (CRF).

### Outcome measurements

Drug efficacy was determined using patient symptoms, body temperature, and virus detection.

#### Primary outcome

The first primary outcome is the duration of the illness and will be measured in hours. Duration of illness is defined as the time from symptom onset to alleviation of the influenza-like symptoms: nasal obstruction, running nose, cough, sore throat, headache, fatigue, myalgia, chills and sweating. The definition of symptom alleviation is influenza-like symptoms score ≤1 (mild) and maintenance of stable symptoms for more than 24 h [30,32].

Another primary outcome is the time to defervescence, which is measured in hours. The time from the first dose of the study medication to the time when the body temperature decreases to <37.4°C is defined as ‘defervescence,’ and when it is sustained for ≥24 h, it is defined as ‘complete defervescence’ [30,32].

#### Secondary outcome

Five secondary endpoints are being considered. The first is the duration of viral shedding. This endpoint is defined as the time from illness onset to the first time the viral nucleic acid test is negative. The second endpoint is disease severity. It is assessed by an area under the curve (AUC) analysis of nine influenza-like symptom scores [29]. The AUC is calculated as the product of the daily symptom scores multiplied by the duration of illness. The third endpoint is the frequency of acetaminophen usage. The fourth endpoint is the prevalence of secondary influenza complications. These are otitis, bronchitis, pneumonia, nasosinusitis, suppurative tonsillitis, acute parotitis, Reye’s syndrome, central nervous system disease, myocarditis, pericarditis, acute myositis, and toxic shock syndrome. The final endpoint was an economic evaluation.

#### Safety evaluation

Safety is evaluated using vital signs, cardiopulmonary signs, adverse reactions, electrocardiography, and clinical laboratory tests (including liver and kidney functions and myocardium enzyme). These indices are compared before and after the drugs were taken.

#### Evaluation of adverse events

An adverse event refers to any adverse, unintended or unplanned effects on vital signs, symptoms, diseases, or laboratory indices that change after participants have enrolled in the clinical trial. An adverse event is not necessarily related to the drugs. Adverse events are divided into three levels: mild, moderate and severe (Table 4). The cause-and-effect relationship between an adverse event and study drugs is evaluated according to whether the agent (i) causes death; (ii) imperils life; (iii) leads to hospitalization or extends the duration of the hospital stay; (iv) is teratogenic or causes birth defects; (v) causes a permanent handicap; or (vi) is carcinogenic (Table 5). All serious adverse events must be reported within 24 h to the State Food and Drug Administration as well as the ethics committee.

**Table 4:** **Classification of disease severity**

**Classification**	**Features**
**1. Mild**	Can continue to participate in the clinical trial, without obstacles in the activities of daily living
**2. Moderate**	Has certain obstacles to daily life, but not to the point of completely diminished function
**3. Severe**	The extent of obstacles in the activities of daily living has led to the risk of permanent disability, and appropriate measures are needed to mitigate medical issues

**Table 5:** **Classification of adverse events**

**Classification**	**Feature**
**1. Definite**	Use of the experimental drug has a definite relationship with time.
	Similar pharmacologic effects of the drug or experimental drug are well known.
	There is no other plausible cause owing to disease or other explanations.
**2. Probable**	Use of the experimental drug has a reasonable relationship with time.
	Similar pharmacologic effects of the drug or experimental drug are well known.
	It is difficult to identify a cause owing to disease or other explanations.
**3. Possible**	Use of the experimental drug has a reasonable relationship with time.
	The adverse event may be caused by disease or other explanations.
**4. Remote**	There is a connection between time and test drug.
	It is easy to explain through disease or the main causes of a disease.
**5. Unrelated**	There is no connection between time and the test drug.
	The adverse event is definitely caused by other reasons, and not the test drug.

### Data management

#### Management of the analyses of data entry by a third party (Southern Medical University, China)

Management of the data entry and analyses will be undertaken by a specific data manager responsible for building the study database and program settings. Data will be inputted twice and confirmed by two keyboard operators who have received special training. After verification of the case report forms (CRFs), identified input errors will be corrected until there are no differences in the database. Uncertainties can be discussed with the researcher using CRF question forms. These question forms shall be completed by the researcher within a specified time limit as well as being signed and dated. Completed question forms will be sent to the Department Of Database Management. The data manager will revise and verify the feedback from the researcher, update the database, and resend the form (if necessary). After confirming the veracity of the database *via* blind review, locked data will not be corrected. Data revision after locking will be confirmed by the study promoters, researchers, program manager, statistician, and the data manager with a written declaration and will be corrected in the statistical analyses. After data review, all cases will be evaluated by confirming the intent-to-treat population (ITTP), modified intent-to-treat population (MITTP), per-protocol population (PPP) and safety analysis population (SAP). The decision will be approved by the data manager, study promoter, and researchers, and will end with unblinding after locking the database.

#### Patient groups for data analyses

##### Intent-to-treat population

The intent-to treat population will include those patients who agree to enroll in the study and sign an informed consent form.

##### Modified intent-to-treat population

The modified intent-to-treat population includes those patients who used the test drug at least once during randomization, and data will be used to indicate efficacy after intervention.

##### Per protocol population

The per protocol population includes those cases that meet the inclusion criteria, are in full accordance with the test program (or violate the protocol only slightly), complete the trial and complete the CRF. The PP is used to analyze the main indicators for evaluating efficacy and to examine the consistency of the results from the MITT.

##### Safety analysis population

For the safety analysis population, data will demonstrate efficacy after intervention for those patients who use the test drug at least once during randomization. In this study, the baseline data, MITT analyses and main indicators for treatment are employed in PP analyses, but the conclusion from MITT analyses is also evaluated. When undertaking PP analyses, if there are missing data, the last observation carried forward (LOCF) approach will be adopted to complete the dataset. For laboratory data, adverse events and side effects, we will use SA analyses, and the SAP will be the denominator for the prevalence of adverse reactions.

#### Statistical analyses

The software used for statistical analyses will be SPSS v17.0 (IBM, Armonk, NY, USA) or SAS v9.2 (SAS Institute, Cary, NC, USA).

All statistical inferences will be determined using two-sided tests. *P* <0.05 will be considered significant, and 95% confidence intervals will be used.

Efficacy analyses will use LOCF to compensate for cases not fully covered during treatment. The LOCF approach uses the last data observed, which is transferred to the database to obtain a complete dataset. The safety assessment will not evaluate missing data.

A comparison between the dropout rate and dropout rate caused by adverse events will be undertaken using the Pearson’s χ^2^test.

Data will be described as the mean, standard deviation, and confidence intervals. If necessary, minimum, maximum, P25, P75, and median values will be provided. Paired measurement data will also show the differences between the mean and standard deviation. When using nonparametric methods, median and mean values will be provided. Count data will be described using the frequency distribution and corresponding percentage. Data of concentrations will be described using the frequency distribution and the corresponding percentage, as well as median and average values. Qualitative information will be described using the positive rate, the number of positive cases, and the denominator.

Baseline data analyses (two sets) will include demographic indicators, as well as general, primary and secondary indicators before intervention. Measurement data will be described using a *t*-test or t’ test (if the variance is absent). Count data will be described using the Pearson’s χ^2^test. Rating data will be described using the two-sample Wilcoxon rank sum test.

With respect to analyses of effectiveness, for quantitative variables, comparisons between groups will be undertaken using repeated measures analysis of variance and covariance analysis. For qualitative variables, comparisons between groups will be tested using the Pearson’s χ^2^test, whereas center effect analysis will use CMH. For rating variables, comparisons between groups will be tested using the Kruskal-Wallis test, regression analysis center effect via the CMH test, or grade logistics.

In terms of center effect analyses, the GLM method will be used for quantitative indicators and CMH methods for qualitative indicators. Rating variables will be evaluated and corrected using a logistic regression model.

With regard to subgroup analyses, depending on the circumstances, factors that cannot be excluded may have an effect on the prognosis.

For safety analyses, the prevalence of adverse events in the two groups will be compared using the Pearson’s χ^2^test, as well as listing and describing the events that occur during the trial. A description of laboratory test results before and after the test will be described as normal/abnormal changes, as well as the relationship between the abnormal changes and the test drug; these changes will be stated.

### Safety and ethics

The drugs used in this clinical trial (BLG granules and oseltamivir) have been approved for clinical use, so there is no potential safety hazard. To ensure that study patients receive effective treatment during rehabilitation, the placebo control group will be provided basic drugs.

Before study commencement, we will explain in detail the research purpose, methods and processes. Satisfactory answers to any questions from the subjects, after obtaining written informed consent, will be included. Also, the center will focus on the purpose, application and execution of the study. Researchers will accept the research plan, treatment procedure, patients, research schedule, CRF and written informed consent. During the trial, the clinical unit must comply with GCP and the scheme of the clinical trial. Pharmaceutical supervisory and administrative departments will occasionally conduct audits and inspections and will cooperate with the sponsor to send medical examiners. Any moderate or serious adverse events will be reported to the Ethics Committee.

## Discussion

Treatment effectiveness, safety assurance and patient affordability are the key factors for any drugs, including Western medicines and TCHMs. This projected clinical trial will provide evidence as to whether BLG granules are suitable and safe for treating and preventing seasonal influenza.

BLG seems to display a clinical effect during the early onset of illness. Our previous study showed that *IPRS*, clemastanin B and lignin, which are separated from *IIR*, have antiviral roles in the early infection stage of the influenza virus [24-26]. Accordingly, we defined the time of illness onset to be <36 hours. Unfortunately, this criterion made patient recruitment quite difficult. However, this design, which is based on a combination of traditional medical experience and modern pharmacological study, should allow us to discover the objective treatment effectiveness of BLG.

The safety of TCHM is attracting major attention. First, in our study, BLG granules are used only for 5 days in the acute phase, so that it may be safe for humans. Second, we evaluate the safety of BLG based on changes in liver and kidney function after treatment. In addition, western medicines used for treating influenza (such as oseltamivir, which sells for 283 RBM/box in China) is expensive for ‘developing’ countries. Hence, we need to explore new, safe and inexpensive anti-influenza medicine. BLG, which sells for 12.00 RBM/box in China, would be a good choice.

Furthermore, according to basic science studies, the active ingredient of *IIR* also exerts immune-modulating and anti-inflammation effects *in vitro* and *in vivo*. Polysaccharides from *IIR* can promote production of interleukin-2 and interferon-γ, as well as the proliferation of lymphocytes and macrophages in mice [33]. Also, indirubin and its derivatives can suppress the expression of several pro-inflammatory cytokines induced by influenza infection in human macrophages and alveolar epithelial cells [27]. Hence, an evidence-based clinical trial to evaluate the effectiveness of BLG in treating inflammation induced by influenza infection certainly has merit.

In summary, we have described the first clinical trial for treatment using a single herb against influenza A and B viruses in China. We will hold a large-scale clinical trial to comprehensively evaluate the effectiveness and safety of BLG against influenza infection based on the results of this pilot study.

## Trial status

This clinical trial was reviewed by the Ethics Committee of the First Affiliated Hospital of Guangzhou Medical University at the end of 2011. The first patient was enrolled in 2012. From early 2012 to 2014 in June, 50 patients had finished the treatment and observation cycle. This clinical trial is expected to be completed at the end of 2015.

## Additional files

Additional file 1:
**The product inspection report of Ban-Lan-Gen (the Chinese edition).** The Ban-Lan-Gen (BLG) granules were produced by *Hutchisom Whampoa Guangzhou Baiyunshan Chinese Medicine Company Limited*. The items, including shape and properties, solubility, bacterial count, *etcetera* of the BLG granules, were tested, and they conformed to the quality requirements of the *People’s Republic of China Pharmacopoeia* of 2010. Additional file 2:
**The product inspection report of placebo (the Chinese edition).** The placebo was produced by *Hutchisom Whampoa Guangzhou Baiyunshan Chinese Medicine Company Limited*. The items, including shape and properties, solubility, bacterial count, *etcetera* of the placebo, were tested, and they conformed to the quality requirements of the *People’s Republic of China Pharmacopoeia* of 2010.

## Abbreviations

AUC: area under the curve
BMI: body mass index
CRF: case report form
CK: creatine kinase
CK-MB: creatine kinase isoenzyme
GCP: good clinical practice
HA: hemagglutinin
IIR: *Isatis indigotica* root
ILI: influenza-like illness
IRPS: *Isatis indigotica* root polysaccharide
ITTP: intent-to-treat population
LOCF: last observation carried forward
MITTP: modified intent-to-treat population
NA: neuraminidase
PPP: per-protocol population
SAP: safety analysis population
TCHM: Traditional Chinese herbal medicine
