# Supplementary material for: Efficacy and safety of Ban-Lan-Gen granules in the treatment of seasonal influenza: study protocol for a randomized controlled trial
Source: Trials. 2015 Mar 28;16:126. doi: 10.1186/s13063-015-0645-x (PMC4383212; doi:10.1186/s13063-015-0645-x)
Supplement: Additional file 1: — The product inspection report of Ban-Lan-Gen (the Chinese edition). The Ban-Lan-Gen (BLG) granules were produced by Hutchisom Whampoa Guangzhou Baiyunshan Chinese Medicine Company Limited. The items, including shape and properties, solubility, bacterial count, etcetera of the BLG granules, were tested, and they conformed to the quality requirements of the People’s Republic of China Pharmacopoeia of 2010. [file 13063_2015_645_MOESM1_ESM.pdf]

广州白云山和记黄埔中药有限公司 HWL

广州白云山和记黄埔中药有限公司 质量控制中心

成品检验报告书

控制号：C1412-1001

检品名称	板蓝根颗粒	代号	
检品来源	研究院	批号	140225
检品数量	20小袋	规　格	10g/袋
检验项目	全检	收样日期	2014.03.12
检验依据	中国药典2010年版第二增补本	报告日期	2014.03.17

检验项目	法定标准	检验结果
性状:	本品为浅棕黄色或棕褐色的颗粒.味甜,微苦。	符合规定
鉴别:	与板蓝根对照药材、亮氨酸、精氨酸对照品一致。	符合规定
溶化性:	5分钟内全溶，应无焦屑等异物	符合规定
水分:(%)	≤6.0	0.8
粒度: (%)	≤15.0	2.4
装量差异: (%)	±5	符合规定
细菌数: (cfu/g)	≤1000	30
霉菌和酵母菌数: (cfu/g)	≤100	<10
大肠埃希菌:	每1g不得检出	未检出

结论：本品按中国药典2010年版第二增补本检验,结果符合规定。

QC经理：欧阳惠芳　　复核人：汪许敏 冯多仪　化验员:赖桂英 张瑞雪
