# Supplementary material for: Efficacy and safety of Ban-Lan-Gen granules in the treatment of seasonal influenza: study protocol for a randomized controlled trial
Source: Trials. 2015 Mar 28;16:126. doi: 10.1186/s13063-015-0645-x (PMC4383212; doi:10.1186/s13063-015-0645-x)
Supplement: Additional file 2: — The product inspection report of placebo (the Chinese edition). The placebo was produced by Hutchisom Whampoa Guangzhou Baiyunshan Chinese Medicine Company Limited. The items, including shape and properties, solubility, bacterial count, etcetera of the placebo, were tested, and they conformed to the quality requirements of the People’s Republic of China Pharmacopoeia of 2010. [file 13063_2015_645_MOESM2_ESM.pdf]

广州白云山和记黄埔中药有限公司 HWL

广州白云山和记黄埔中药有限公司 质量控制中心

# 成品检验报告书

控制号：C1412-1003

| 检品名称 | 板蓝根颗粒安慰剂 | 代号 | |
|---|---|---|---|
| 检品来源 | 研究院 | 批号 | 140226 |
| 检品数量 | 20袋 | 规　格 | 10g/袋 |
| 检验项目 | 全检 | 收样日期 | 2014.03.12 |
| 检验依据 | 参考中国药典2010年版第二增补本 | 报告日期 | 2014.03.17 |

| 检验项目 | | 检验结果 |
|---|---|---|
| 性状: | 本品为棕褐色的颗粒，味甜。 | 符合规定 |
| 鉴别: | 与板蓝根对照药材溶液、亮氨酸、精氨酸对照品一致。 | 未检出 |
| 溶化性: | 5分钟内全溶，应无焦屑等异物 | 符合规定 |
| 水分:(%) | 不得大于6.0 | 0.9 |
| 粒度:(%) | 不得大于15.0 | 1.7 |
| 装量差异:（%） | ±5 | 符合规定 |
| 细菌数:（cfu/g） | ≤1000 | 30 |
| 霉菌和酵母菌数:（cfu/g） | ≤100 | 10 |
| 大肠埃希菌: | 每1g不得检出 | 未检出 |

结论：本品参考中国药典2010年版第二增补本标准检验,结果如上。

QC经理：欧阳惠芳　　复核人:汪许敏 冯多仪　　化验员:江红苑 张瑞雪
